# Antimicrobial susceptibility pattern, and associated factors of Salmonella and Shigella infections among under five children in Arba Minch, South Ethiopia

**DOI:** 10.1186/s12941-018-0253-1

**Published:** 2018-02-01

**Authors:** Gemechu Ameya, Tsegaye Tsalla, Fasil Getu, Eyob Getu

**Affiliations:** 1 Department of Medical Laboratory Science, College of Medicine and Health Sciences, Arba Minch University, P.O. Box: 21, Arba Minch, Ethiopia; 2 Department of Medical Laboratory Science, Harar Regional Blood Bank, Harar, Ethiopia; 3 Department of Medical Laboratory Science, Yirgachefe primary Hospital, Dilla, Ethiopia

**Keywords:** Antimicrobial activity, Diarrhea, Salmonella, Shigella, Under five children

## Abstract

**Background:**

Diarrheal diseases continue to be the major cause of morbidity and mortality among children under 5 years. *Salmonella* and *Shigella* specious are the major enteric pathogen causing diarrhea among children worldwide. Examination of stool sample is the most sensitive method to diagnose diarrheal disease in children. This study aimed to determining the prevalence, antimicrobial susceptibility pattern and associated factor of Salmonella and Shigella infection among under five children.

**Methods:**

A cross sectional study was conducted on under 5 years children attending Arba Minch town. Pre-tested and structured questionnaire was used for collecting data about socio-demographic characteristics and associated factors. Stool sample was used to isolate and identified the pathogen. Antimicrobial susceptibility test was performed for isolated *Salmonella* and *Shigella* specious. A logistic regression analysis was used to see the association between different variables and outcome variable. Odds ratio with 95% CI was computed to determine the presence and strength of the association.

**Results:**

A total of 167 under five children were included in the study. About 57% of participants were males with the mean age of 32 months. The overall prevalence of *Salmonella* and *Shigella* species infection was 17.45% with 12.6% *Salmonella* species. The isolates were resistant to common antibiotics such as Amoxicillin, Erythromycin, Chloramphenicol, Clindamycin, Norfloxacin, Ciprofloxacin, Cotrimoxazole, and Gentamycin. Urban resident [AOR = 7.11; 95% CI (2.3, 22.2)], month income < 1000 Ethiopian birr [AOR = 6.5; 95% CI (2.0, 21.4)], absence of waste disposal system [AOR = 3.3; 95% CI (1.2, 9.3)], poor hand washing habit [AOR = 6.0; 95% CI (2.0, 18.2)], untrimmed finger nail [AOR = 3.7; 95% CI (1.4, 10.6)], and use of napkin [AOR = 3.2; 95% CI (1.1, 9.3)] had significant association with Salmonella and Shigella infection.

**Conclusion:**

*Salmonella* and *Shigella* species infections were higher as compared the national prevalence. This study also revealed that the enteric infection were significantly associated with finger nail status, residence, hand washing practice, month income of parents, usage of napkin after toilet, and absence of waste disposal system. Therefore, working on identified associated factors and regular drug susceptibility test is mandatory to reduce the problem.

## Background

Diarrhea is a worldwide problem that frequently encountered in the practice of pediatric medicine. Globally 550 million people annually become ill. Of these 220 million of cases occur in children under the age of 5 years [1]. According to the world health organization report diarrheal illness is also the second leading causes of death in children younger than 5 years. Globally 21% of deaths in children under the age of 5 years results from diarrheal infection [2]. Diarrhea kills more young children than Malaria, AIDS, and Measles combined [3]. In developing countries, *Shigella* and *Salmonella* species remain major contributors to acute enteric infection in children. Asia, Africa and Latin America had an estimated 2.5 million deaths each year in children under the age of five [4]. In persist cases it can cause dehydration due to large amount of fluid loss which often begin with loss of the normal stretchiness of the skin and irritable behavior. Further complication may result in decreased urination, loss of skin color, and fast heart rate [5, 6].

Diarrhea is mainly caused by infection with virus, bacteria or parasite [4]. Diarrhea caused by parasite has slow onset of action unlike that of bacterial or viral infection. Under normal condition, the gastrointestinal tract has great capacity to absorb fluid and electrolyte. However, enteric pathogens disturb this balance by different mechanisms. All this action results in fluid and electrolyte loss and this may leads to death [7]. Bacterial diarrhea is commonly caused by *Salmonella enterica*, *Shigella* species, *Vibrio cholera*, *Clostridium difficile*, *Escherichia coli*, *Campylobacter jejuni* and others. Among these *Salmonella* and *Shigella* specious are the endemic in most part of developing countries [4].

Salmonella causes Salmonellosis which can be characterized by diarrhea, fever, vomiting and abdominal cramps after 12–72 h of infection. *Salmonella enteric* serotype typhi is the common serotype of Salmonella that causes of typhoid fever. Typhoid fever is a systemic disease with diarrhea and it is the major causes of morbidity and mortality worldwide in under the age of five children [8]. Shigellosis is also known as bacillary dysentery or Marlow syndrome is caused by *Shigella* species and it can rarely occur in animals other than humans [9]. In addition *Shigella dysenteriae* species releases Shiga toxin, an AB exotoxin similar to Entero EHEC that cause diarrhea [10].

Different factors like socio-economic characteristic of the patient, poor access to latrine, lack of clean drinking water can be associated with diarrhea diseases. Poverty is a good indicator of acute children diarrhea. It is associated with poor housing, crowding, dirty floor, lack of clean water and poor food storage condition. It can also be associated with cohabitation with domestic animals [11]. Laboratory diagnosis of Salmonella and Shigella are mainly performed by culturing of the organism or through the demonstration of specific antibody or antigen in the serum or urine. Now a days, multiple drug resistant *Shigella* and *Salmonella* species are frequently isolated in clinical samples. This makes them difficult to be treated. Antimicrobial resistance within wide range of infectious agents is growing public health treat of the twenty-first century [12].

Nowadays different researches conducted in Ethiopia showed that multiple drug resistant *Salmonella* and *Shigella* species are isolated from children under the age of five [13]. There is limited published research on prevalence, antimicrobial susceptibility pattern and associated factor of Salmonella and Shigella infection among under the age of five children in southern part of Ethiopia. Therefore, this study is aimed to determine the prevalence, drug sensitivity pattern and associated factors of *Shigella* and *Salmonella* species infections among under five children in Arba Minch town Governmental Health Institutions, southern Ethiopia.

## Materials

### Study design, setting, and period

An institutional based cross sectional study was conducted on governmental health institute on antimicrobial sensitivity and factor associated with Salmonella and Shigella infections among under the age of five children. The study was conducted in Arba Minch town governmental health institutes, southern part of Ethiopia. The study was conducted from March to May, 2017.

### Source population and sampling process

Under five children attending at the three governmental health institutions of Arba Minch town (Arba Minch General Hospital, Shecha Health Center, and Sikela Health Center) during study period were source population. Systematic sampling method was used to select 167 under five children with diarrhea disease who fulfill inclusion criteria during the study period using case interval. The first case of the interval was selected by lottery method. All under five children with diarrhea disease were included in the study. Patients who had received antibiotic treatment within 1 week for their symptom before coming to hospital were excluded from the study.

### Data collection

A pre-tested, structured questionnaire was used to collect socio-demographic, socio-economic and clinical data of the children. The samples were proportionally allocated in each health institutions. Information related to children age, sex, family income, family size, educational status of the parent/guardian, family occupation, latrine usage, hand washing habit before having meal, hand washing habit after visiting toilet, hand washing habit after changing napkins, availability of dry and liquid waste disposal system, feeding practice, vegetable washing habit, and finger nail status.

### Stool sample collection

Parent or care giver of children was informed to bring stool sample of their children. A clean, dry and leak proof stool cup and clean wooden applicator stick were given for stool specimen collection. The children attendant was informed to bring approximately 2 g of stool. After collection the stool sample was transported to Arba Minch University College of Medicine and Health Science, Microbiology Laboratory within 1 h.

### Stool examination

First physical examination of the stool sample was performed in order to check whether the sample was diarrheal or not, presence of blood, pus and mucus. Immediately after physical examination of fecal specimen, direct microscopic examination was performed. This involves reporting of the appearance of the specimen and identifying any parasitic larva, ova, cyst, and trophozoite of some parasites microscopically.

### Culturing and identification

The collected stool specimen was processed for bacteriological analysis. The stool sample was inoculated into MacConkey (Oxoid Ltd.) and Xylose Lysine Deoxycholate agar (Oxoid Ltd.) by using sterile wire loop. The inoculum was incubated under aerobic condition at 37 °C for 24 h. After period of incubation, the plates were examined for colony characteristics of *Salmonella* and *Shigella* specious. Colorless to yellow colonies on MacConkey agar, and Pink to red colonies on Xylose lysine deoxycholate agar were further identification of species of pure isolates by biochemical tests [14].

### Antimicrobial susceptibility testing

In vitro antimicrobial susceptibility test was carried out for identified *Salmonella* and *Shigella* species. The susceptibility test was performed on Muller Hinton agar by using Kirby–Bauer disc diffusion technique. Precisely, pure identified colonies from the overnight culture was suspended in nutrient broth and incubated for 4 h at 37 °C. Turbidity of broth culture was checked against 0.5 McFarland standards. By using sterile swab the organism in broth was uniformly inoculated into Muller Hinton agar. The antibiotic discs were applied on the surface of the inoculated agar. Antimicrobial disc were selected according to committee for clinical laboratory standard (CCLS), 2015 list of drug for specific isolated pathogen and based on locally availability of the drug. The drugs used in the antibiotic susceptibility test are Amoxicillin, Erythromycin, Chloramphenicol, Clindamycin, Norfloxacin, Ciprofloxacin, Cotrimoxazole, and Gentamycin. After overnight incubation, the diameter of growth inhibition around the discs was measured and interpreted as sensitive, intermediate or resistant according to clinical and laboratory standards institute (CLSI) [15]. Known *Salmonella* and *Shigella* species were used as positive control in all methods.

### Data processing

Collected data was entered to Epi-Info version 7.2.1 and exported to SPSS version 20.0 for further analysis. Descriptive statistics and logistic regression were used for analysis of data. Logistic regression analysis was used to look into the association between associated factors and bacterial enteric infection. Odds ratio (OR) and 95% confidence interval (CI) were used to determine significance between *Salmonella* and *Shigella* specious and different variables. Whose variables with level statistically significant P < 0.25 on Bivariate analysis were entered jointly into a multivariate logistic regression. *P* value less than 0.05 was considered as statistically significant.

## Results

### Socio-demographic characteristics

A total of 167 children with diarrhea were included in our study in which study participants were proportionally allocated for the three Arba Minch governmental health institutes particularly Arba Minch general hospital, Sikela health center and Secha health center. The age of participants range between 5 and 59 months and mean age was 32 months. Of all the study participants, 43.1% were females and nearly half of the participants were urban dweller. About one-fourth of caregivers or parents of the children were illiterate while one-third of them learned primary education. One-fourth of the children were single parent. Among all caregivers’ or parents’ of the children 33% were housewife (Table 1).

**Table 1:** Socio-demographic status of study participants

Variables	Frequency	Percent
Sex		
Male	95	56.9
Female	72	43.1
Age (in year)		
< 1	33	19.8
1–3	65	38.9
3–5	69	41.3
Resident		
Urban	80	47.9
Rural	87	52.1
Educational status of attendants		
Illiterate	45	26.9
Primary education	55	32.9
Secondary education	41	24.6
College and above	26	15.6
Marital status		
Unmarried	19	11.4
Married	126	75.4
Divorced	16	9.6
Widow	6	3.6
Monthly income (Ethiopia Birr)		
< 500	36	21.6
500–1000	47	28.1
1000–1500	47	28.1
> 1500	37	22.2
Family size		
< 3	29	17.4
3–5	64	38.3
> 5	74	44.3
Occupation		
Housewife	56	33.5
Private	52	31.1
Government employer	36	21.6
Student	17	10.2
Other	6	3.6

### Magnitude of enteric pathogen

Of 167 diarrheic children, *Salmonella* species was detected in 12.6% [95% CI (8.6–16.60)] study participants and *Shigella* species was detected in 4.8% [95% CI (2.1–7.5)] children. About one-third of detected Salmonella cases were in infants and *Shigella* species were more detected in age group between 3 and 5 years. Among 29 exclusive breast feeding children, *Salmonella* species was isolated in 5 (17.2%) of them and one Shigella case was also observed. Urban dwellers were more infected with the enteric pathogen than rural resident. Among children of illiterate parents, the magnitude of the enteric pathogens was one-fifth the children.

### Antimicrobial susceptibility test

All isolated *Salmonella* species were not sensitive for Ampicillin and Erythromycin. The isolated enteric pathogens have resistant rate of 48, 44.8, and 34.5% against Clindamycin, Chloramphenicol, and Cotrimoxazole, respectively. Ciprofloxacin was sensitive for majority (89.65) of the isolated enteric pathogen. Norfloxacin and Gentamycin has sensitivity rate of 57.38 and 52.4% against Salmonella isolates, respectively (Table 2).

**Table 2:** Antimicrobial susceptibility patterns of Salmonella and Shigella isolates in under five children in Arba Minch town Governmental Health Institutions

Antibiotics	*Salmonella* species no (%)			*Shigella* species no (%)			Total no (%)		
	S	I	R	S	I	R	S	I	R
AMP	0	0	21 (100)	0	0	8 (100)	0	0	29 (100)
ERY	0	12 (57)	9 (43)	0	3 (37.5)	5 (62.5)	0	15 (52)	14 (48)
CAM	5 (24)	7 (33)	9 (43)	1 (12.5)	3 (37.5)	4 (50)	6 (20.7	10 (34.5)	13 (44.8)
CLI	9 (42.8)	7 (33)	5 (24)	1 (12.5)	4 (50)	3 (37.5)	1 (3.5)	11 (38)	17 (58.5)
NX	12 (57)	8 (38)	1 (4.8)	4 (50)	3 (37.5)	1 (12.5)	13 (44.8)	11 (38)	5 (17.2)
CIP	20 (95.24)	1 (4.76)	0	6 (75)	2 (25)	0	26 (89.65)	3 (10.34)	0
CTX	7 (33)	6 (29)	8 (38)	3 (37.5)	3 (37.5)	2 (25)	10 (34.5)	9 (31)	10 (34.5)
GEN	11 (52.4)	6 (29)	4 (19)	3 (37.5)	3 (37.5)	2 (25)	14 (48)	9 (31)	6 (21)

*S* sensitive, *R* resistant, *I* intermediate, *AMP* Amoxicillin, *ERY* Erythromycin, *CAM* Chloramphenicol, *CLI* Clindamycin, *NX* Norfloxacin, *CIP* Ciprofloxacin, *CTX* Cotrimoxazole, *GEN* Gentamycin

**Fig. 1:**
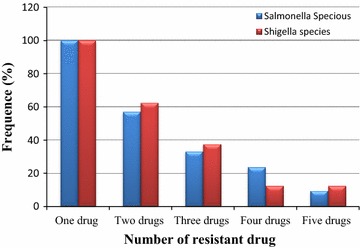
Multi drug resistance pattern of bacterial isolates. [One drug (AMP), two drugs (AMP and ERY), three drugs (AMP, ERY, and CAM), four drugs (AMP, ERY, CAM, and CLI), five drugs (AMP, ERY, CAM, CLI, and NX)]

Resistance for one or more antibiotics was observed among the isolated enteric pathogens. Nearly 60% of isolated pathogens were resistant to two drugs (Ampicillin and Erythromycin). About third of isolated *Salmonella* and *Shigella* species were multi drug resistant. High resistances were observed for Ampicillin and Erythromycin. The resistant rate both species for Ampicillin, Erythromycin and Chloramphenicol was 37.5. The pathogen resistant to four and five drugs were 20.68 and 10.34% of the total isolates, respectively (Fig. 1).

### Factors associated with enteric pathogenic infection

Logistic regression analysis was performed to check whether each independent predictor had significant association with the outcome variable after checking the fitness of the model for the variable by Hosmer and Lemeshow. Those predictors that were identified by bivariate analysis were adjusted by using multivariable backward stepwise logistic regression. Binary logistic regression of the socio-demographic, medical condition, and associated factors variables showed that age, marital status, residence, monthly income, occupation, finger nail length, hand washing habit, availability of latrine, usage of napkin, and availability of west disposable site were transferred into multivariable logistic regression.

Among variables transferred into multivariable logistic regression, finger nail trimming status, residence, hand washing habit, availability of waste disposal system, month income, and usage of napkins had significant association with Salmonella and Shigella infection of under five children. Those children who live in urban have about seven times more chance of infection with Salmonella or Shigella than those children who live in rural area [AOR = 7.11; 95% CI (2.3, 22.2)]. Parent with month income less than 1000 Ethiopian birr have about 6.5 odd of infection of their under five children with the enteric pathogen than parent with more than 1000 birr [AOR = 6.5; 95% CI (2.0, 21.4)]. Children who live in environment without waste disposal system have about three times chance of infection with Salmonella/Shigella than those children from proper west disposal environment [AOR = 3.3; 95% CI (1.2, 9.3)]. Absence of proper hand washing habit have six times odds of under five children Salmonella/Shigella infection than those who have hand washing habit before meal [AOR = 6.0; 95% CI (2.0, 18.2)]. Children with untrimmed finger nail have about four times chance of getting the enteric infection than children with trimmed finger nail [AOR = 3.7; 95% CI (1.4, 10.6)]. Usage of napkin reduces chance of Salmonella/Shigella infection of under five children by three times [AOR = 3.2; 95% CI (1.1, 9.3)] (Table 3).

**Table 3:** Factors associated with Salmonella and Shigella infections among under 5 years children in Arba Minch Governmental Health Institutions

Variable	Salmonella/Shigella infection	No Salmonella/Shigella	COR (95% CI)	AOR (95% CI)	P-value
Residence					
Urban	21	59	3.5 (1.4, 8.5)	7.11 (2.3, 22.2)	0.001
Rural	8	79	1	1	
Month income (ET Birr)					
< 1000	23	60	4.9 (1.9, 13.0)	6.5 (2.0, 21.4)	0.002
> 1000	6	78	1	1	
Availability of waste disposal system					
Available	11	87	1	1	
Not available	18	51	2.8 (1.22, 6.4)	3.3 (1.2, 9.2)	0.023
Hand washing habit					
Yes	7	83	1	1	
No	22	55	4.7 (1.9, 11.8)	6.0 (2.0, 18.2)	0.001
Finger nail					
Trimmed	10	93	1	1	
Untrimmed	19	45	3.9 (1.7, 9.1)	3.7 (1.4, 10.6)	0.011
Usage of Napkin					
Yes	18	57	2.3 (1.02, 5.3)	3.2 (1.1, 9.3)	0.03
No	11	81	1	1	

## Discussion

In this study a total of 29 (17.45%) positive cultures for Salmonella/Shigella infection from 167 patients were identified. Among the positive cultures, Salmonella was detected in 12.6% and Shigella was detected in 4.8%. This finding is similar to studies conducted in Jimma [16], Addis Ababa [13] and Hawassa [17]. In addition to this, the result of this study was in line with the study conducted in Nepal [18], Turkey [19] and Nairobi [20] where 4.6, 3.2, and 2.5%, respectively. Our study has lower prevalence of Shigella compared to the study conducted in Bahir Dar [21], Mekelle [22], Anyigba [23] and Gaborone [24] with prevalence of 7.85, 13.3, 21.9 and 21% respectively. In contrast, the study conducted in Nigeria–Benin Teaching Hospital, is lower than our finding (1.4%) [25]. The variation may be due to the difference in socio-demographic status, altitude, water supply or population size of the two cities.

The magnitude of *Salmonella* species was 12.6% in the under five children with diarrhea. The finding of this study is similar to the finding of Kenya-Igembe district hospital which found to be 10.4% [26]. A higher prevalence was observed when it is compared with the study conducted in Jimma [16] and Bahir Dar [21] in which prevalence was 6.2 and 7.8%, respectively. However, this result showed an increase in prevalence of Salmonella when compared with the result of Addis Ababa [13], Hawassa [17], Turkey [19], and Kenya [20] with 3.95, 1.5, 3 and 2.5%, respectively. The variation may be due to the socio-economic status, source of drinking water supply, sanitation and hygiene practice of the people in the cites.

In this study the prevalence of Salmonella and Shigella was higher in children between the age group of 1–3. The possible reason may be due to less immune status against the enteric pathogen and lack of effective follow up from their parents/caregiver. About one-third of infected children were from illiterate family. This shows that, most illiterate families didn’t provide good health care for their child than those who educated. In our study the magnitude of enteric pathogens were high in children who feed breast milk alone. This may be due to less number of normal floras that help to prevent pathogens when compared to children who start complementary food. Therefore, the variations may be due to the starting of children to self-feeding that help to develop microbial flora.

This study revealed that factors associated such as residence of the children, month income of the parent, availability of west disposal system of the children living environment, hand washing habit, finger nail status, and usage of napkin associated with Salmonella and Shigella infection among the under five children. Under five children who live in urban area at risk of the enteric pathogen infection This may be due large number of population in urban area and in association with this there is large production of wastes. In our study children came from area were there was no waste disposal system had three times more chance of infection with Salmonella/Shigella infection than those came from area where there is waste disposal system. Studies also showed association between domestic wastes and enteric pathogen infection [27, 28].

Under five children who had untrimmed finger nail have about four times more odds of Salmonella and Shigella infection than those who had trimmed finger nail. Similarly under five poor hand washing habit of parent/caregiver or children had about four times more odds of *Salmonella* and *Shigella* species infection than those who wash their hand before meal. This is in line with the study conducted Igembe district hospital, Kenya which determined hand washing practice before meal as a factor for *Salmonella* and *Shigella* species infections [26]. Unlike this study, a study conducted in Jigjiga, Somali region, Ethiopia revealed that major factors exposing to diarrheal infection were education of the primary caretaker, occupations of the father [29]. The variations may be due to difference in culture, the way of life and socio-demographic characteristics of between the study groups.

Antimicrobial susceptibility test was performed for isolated *Salmonella* and *Shigella* species revealed that both enteric pathogens were not sensitive for Ampicillin. About 42% of Salmonella and 62.5 of Shigella were resistant to Erythromycin. This study is line with the study conducted in Hawassa which revealed that majority of the isolated Salmonella and Shigella showed resistant to Erythromycin [17]. The isolated enteric pathogens were resistant to Chloramphenicol, Cotrimoxazole and Clindamycin with 43, 38 and 24% magnitude respectively. This result is similar to the study conducted in Jimma [16]. Majority of *Salmonella* and *Shigella* species were sensitive Ciprofloxacin. This result is in line with the study conducted in Bahir Dar, North Ethiopia [21]. Multi drug resistance was observed in majority of the isolated Salmonella and Shigella. Similar to this study multi drug resistant strains were observed in the study conducted in Addis Ababa [13], Nepal [18], and Bahir Dar [21].

## Conclusion

The magnitude of Salmonella and Shigella infections among under five children with diarrhea was higher than the national prevalence. This study also revealed that the enteric pathogen infection were significantly associated untrimmed finger nail, urban residence, hand washing practice, waste disposal system, usage of napkin and month income of the parent/caregivers. It is also showed that significant amount of the isolates resist commonly used antimicrobial drugs. Therefore, improving hygiene status of under five children and implementation work on identified associated factors with regular drug susceptibility test is important to reduce the problem.
